# Effect on hygroscopic characteristics of n‐ZnO additions to resin composite

**DOI:** 10.1111/eos.70029

**Published:** 2025-07-04

**Authors:** Abdulaziz Alayed, Nikolaos Silikas, David C. Watts

**Affiliations:** ^1^ Division of Dentistry School of Medical Sciences University of Manchester Manchester UK; ^2^ Department of Restorative Dental Science College of Dentistry King Saud University Riyadh Saudi Arabia; ^3^ Photon Science Institute University of Manchester Manchester UK

**Keywords:** dental restoration, nanoparticles, permanent, polymerization, solubility

## Abstract

This study investigates the effect of adding different amounts of n‐ZnO to model resin‐based composites on their hygroscopic characteristics. Six groups (*n* = 5) were formulated using Bis‐GMA, TEGDMA, UDMA, inert barium glass powder, silica nanoparticles, and varying amounts of n‐ZnO (0–5 wt.%). The photoinitiator system included camphorquinone, diphenyliodonium hexafluorophosphate, and ethyl 4‐(dimethylamino) benzoate. Sorption, solubility, and hygroscopic expansion specimens were prepared following ISO 4049:2019 and immersed in water for 168 days. Sorption and solubility were assessed with an analytical balance, while hygroscopic expansion was measured using a laser scan micrometer. Zn^2^⁺ release was analyzed using ICP‐MS (*n* = 3). Increasing n‐ZnO concentrations significantly reduced sorption and solubility, with the control (0 wt.% n‐ZnO) showing the highest values (28.7 and 2.1 µg/mm^3^) and the 2 wt.% n‐ZnO group showing the lowest (27 µg/mm^3^, −0.4 µg/mm^3^). However, increasing the concentrations of n‐ZnO significantly increased the hygroscopic expansion. The volumetric expansion for the lowest (control) and highest (n‐ZnO at 3 wt.%) was 1.63% and 1.87%, respectively. ICP‐MS revealed progressively higher Zn^2^⁺ release with increasing n‐ZnO concentration, peaking at 675.1 ppb in the 5 wt.% group. Overall, n‐ZnO reduced sorption and solubility while increasing expansion and Zn^2^⁺ release, with all formulations meeting ISO 4049 standards.

## INTRODUCTION

Advances in resin‐based composites (RBCs) have significantly improved their physical and mechanical properties, making them a preferred choice for restorative dentistry among both patients and clinicians. However, further improvements are needed to ensure physical and chemical stability in a moist environment, for example, by addressing their water sorption, solubility, and hygroscopic expansion [1, 2]. It is critical to study these phenomena because long‐term exposure to an aggressive oral environment can cause the filler/matrix interface of a RBC to degrade over time [1, 3].

Water sorption in RBCs is the process of absorbing or adsorbing fluids such as water and saliva, where absorption involves fluid penetration into the material, and adsorption refers to fluid accumulation on its surface [4, 5]. Water sorption can lead to swelling of the material, known as hygroscopic expansion, which may help in the relaxation of internal stresses [2]. Also, expansion generates stress on the interfacial bond, leading to its breakdown. However, it may compensate for the gap formed during polymerization shrinkage [6, 7]. Water absorbed into the polymer network can act as a space occupier, reducing interchain interactions and leading to plasticization. This can negatively impact the hardness and wear resistance of the material [7].

Solubility, on the other hand, is the amount of material that can dissolve in a specific solvent, such as water, under defined temperature and pressure conditions [4, 5]. The polymerization of RBC results in a highly cross‐linked polymer network; however, unreacted monomers remain, and degradation may cause their leaching. Additionally, ions from filler particles and resin byproducts can be released, potentially raising biological concerns, such as irritating soft tissues, stimulating bacterial growth, and triggering an allergic reaction [8].

The extent of degradation is influenced by multiple factors, primarily the matrix composition, with triethylene glycol dimethacrylate (TEGDMA) being more susceptible to enzymatic hydrolysis than bisphenol A‐glycidyl methacrylate (Bis‐GMA) [9]. Also, the degree of conversion plays a crucial role, as ester groups are more vulnerable to an attack in a loosely cross‐linked polymer network [7]. Other contributing factors include stresses induced by shrinkage, the stability of resin–filler interfaces, filler volume and type, and the density of the cross‐linked polymer network [10, 11].

Prolonged contact with a moist environment, such as human saliva, leads to the degradation of RBCs due to the presence of esterases. These enzymes break down ester‐containing monomers such as Bis‐GMA, TEGDMA, and urethane dimethacrylate (UDMA), weakening the RBC. As a result, the material degrades, increasing its surface roughness and promoting bacterial accumulation, ultimately compromising its longevity [9, 12, 13].

To enhance the material's resistance to bacterial invasion and to extend its lifespan, researchers have studied the incorporation of antibacterial agents such as zinc oxide nanoparticles (n‐ZnO) into RBCs [14, 15]. Zinc oxide, as an inorganic agent, offers the advantages of stability, robustness, and a long shelf life. Additionally, when added to RBC, it will maintain a tooth‐like color, but the material will become opaque, affecting its depth of cure [16].

While RBCs with n‐ZnO have been studied for their antibacterial, mechanical, physical, and chemical properties, their impact on long‐term water sorption and solubility remains insufficiently explored [14, 15, 16, 17, 18, 19, 20, 21]. Additionally, this study employs a trimodal resin monomer system combined with an advanced binary photoinitiator and conventional radiopaque silicate fillers, making it important to see how the material performs.

This study aimed to evaluate the impact of adding n‐ZnO on the hygroscopic characteristics (water sorption, solubility, hygroscopic expansion, and Zn^2^⁺ ion release) of RBCs. The null hypotheses were that there would be no significant difference between the group without n‐ZnO and those containing n‐ZnO in: (1) water sorption and solubility, (2) hygroscopic expansion, and (3) zinc ion release.

## MATERIAL AND METHODS

### Study design

**FIGURE 1:**
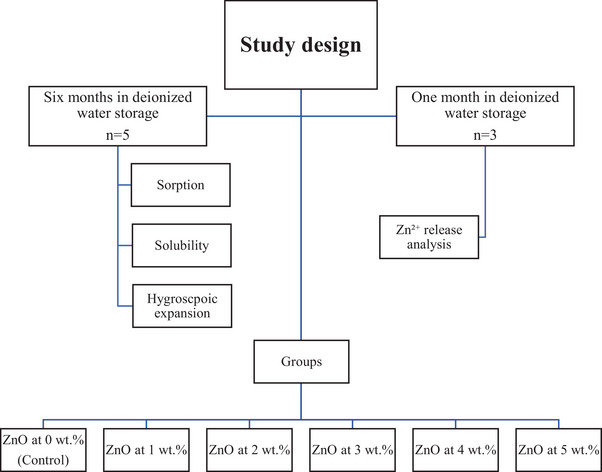
Study design.

Figure 1 shows the study design of adding n‐ZnO to formulated RBCs on the hygroscopic characteristics. The goal was to understand the long‐term effect of adding n‐ZnO on water sorption, sorption, solubility, and hygroscopic expansion. Also, the release of Zn^2^⁺ ions was studied over a period of a month. The independent variable is the ZnO concentration. Dependent variables are water sorption, solubility, hygroscopic expansion, and Zn^2^⁺ ion release.

### Specimen preparation

Six RBCs were formulated, consisting of a 40 wt.% mixture of monomers (40 wt.% Bis‐GMA, 23 wt.% TEGDMA, and 37 wt.% UDMA) and 60 wt.% fillers (55 wt.% inert barium glass powder and 5 wt.% silica nanoparticles). ZnO‐nanoparticles were added at 0 (control), 1, 2, 3, 4, and 5 wt.%. The photoinitiator system consisted of 0.25 wt.% camphorquinone (CQ), 0.25 wt.% diphenyliodonium hexafluorophosphate (DPI), and 0.75 wt.% ethyl 4‐(dimethylamino)benzoate (EDMAB), which were added to the monomer mixture. Further details about the materials used and the groups are presented in Tables 1 and 2.

**TABLE 1:** Chemical components used to formulate the resin‐based composites investigated.

Material	Abbreviation	Type	Particle size & composition	Product or lot no.	Manufacturer
Inert barium glass powder (silanated)	BaSiO_2_	Powder	1.5 µm 55% SiO_2_ 25% BaO 10% B_2_O_3_ 10% Al_2_O_3_	GM27884	Schott
Silicon dioxide (silanated)	SiO_2_	Nanoparticles	15 nm	11‐07	M k Impex
Zinc oxide nanoparticles	n‐ZnO	Antibacterial agent	<100 nm	544,906	Sigma–Aldrich,
Bisphenol glycidyl methacrylate	Bis‐GMA	Monomer	–	811‐39	Esschem Europe
Triethylene glycol dimethacrylate	TEGDMA	Monomer	–	807‐32	Esschem Europe
Urethane dimethacrylate	UDMA	Monomer	–	807‐06	Esschem Europe
Ethyl 4‐(dimethylamino) benzoate	EDMAB	Tertiary amine synergist	–	E24905/MCL5530	Sigma–Aldrich
Diphenyliodonium hexafluorophosphate	DPI	Type I photoinitiator	–	548,014/BCBX5201	Sigma–Aldrich
Camphorquinone	CQ	Type II photoinitiator	–	124,893/09003AQV	Sigma–Aldrich

**TABLE 2:** Composition of formulated RBCs. All groups contained the same amount of photoinitiator system CQ/DPI/EDMAB: 0.25 wt%/0.25 wt%/0.75 wt% of the monomer mixture.

	Monomer and photoinitiator mixture		n‐ZnO		
Groups (wt.%)	Wt %	Vol %	Wt %	Vol %	DC% [16]
Control (ZnO at 0 wt.%)	40	59.4	–		77.2
ZnO at 1 wt.%	39	58.6	1	0.3	76.2
ZnO at 2 wt.%	38	57.8	2	0.6	79.4
ZnO at 3 wt.%	37	57	3	0.9	–
ZnO at 4 wt.%	36	56.2	4	1.2	–
ZnO at 5 wt.%	35	55.3	5	1.6	–

*Note*: The inorganic fillers: 55 wt.%/ ∼ 37 vol% inert barium glass powder and 5 wt.%/ ∼ 4 vol% silica nanoparticles. Degree of conversion (DC) data are provided for up to 2 wt.% [16].

An analytic balance (BM‐252, A&D) was utilized to weigh the materials, and a SpeedMixer (DAC 150.1 FVZ‐K) was used to mix the materials at 3000 rpm for 5 min for each sequence. The mixing sequences were as follows: monomers > add photoinitiator system > add n‐ZnO > add silica nanoparticles > add inert barium glass powder. The formulated composite was then mixed for 5 min. However, mixing was paused after each minute to avoid heat generation.

### Water sorption and solubility

Thirty specimens (*n* = 5/group) were prepared following the ISO 4049:2019 technique with some modification at 23 ± 1°C [22]. The uncured resin was filled in a 15 × 1 mm cylindrical Teflon mold, with two 0.15 mm‐thick transparent polyester films positioned above and below to ensure uniform thickness. A screw‐type clamping device was then applied and tightened manually until firm resistance was encountered, ensuring consistent compression of the mold and resin, further standardizing the thickness. Then, nine overlapping irradiations were applied to the specimen for each side, top, and bottom, each lasting 5 s, using a light‐curing unit (Bluephase PowerCure (Turbo mode), Ivoclar). The curing unit's radiant emittance was verified before curing each specimen using a calibrated radiometer, which was ∼2000 mW/cm^2^ (MARC‐LC, Blue‐Light Analytics). After curing, the specimen edges were finished using 1000‐grit abrasive paper to remove any irregularities.

Finished specimens were first placed in a desiccator containing freshly dried silica gel and incubated at 37°C for 22 h. Then, they were transferred to a second desiccator at 23 ± 1°C for 2 hours. After that, the baseline mass (m_1_) of each specimen was recorded using an analytical balance (BM‐252, A&D). This was done by weighing them repeatedly to an accuracy of 0.1 mg, ensuring a constant mass with no loss greater than 0.1 mg over 24 h. The weight of the specimens stabilized after 2 weeks and 3 days. Each specimen was separately immersed in 10 mL of deionized water and stored in an incubator at 37 ± 1°C. Then, they were removed at specific intervals (1 h, 3 h, and 1, 2, 3, 4, 5, 6, 7, 14, 21, 56, 84, 112, 140, and 168 days) to be measured on the analytical balance. After removal, visible moisture was removed by air‐blowing and waving for 15 s, followed by a 1‐min wait before recording the mass. Each time a specimen was removed and measured, its mass was recorded as (m(t)). The final saturated mass, measured after 168 days, was denoted as (m₂). Specimens were then reconditioned to constant mass (m_3_) in desiccators for 168 days using the same cycles as mentioned before when immersing them in water.

To calculate mass change (%), sorption, and solubility, the following formulas were used:

(1) $$\mathrm{mass}\;\mathrm{change}\;\left(\%\right)=\frac{m\;\left(t\right)-m_{1}}{m_{1}}\;\times 100$$

(2) $$\mathrm{Sorption}\;\left(\%\right)=\frac{m_{2}-m_{3}}{m_{1}}\;\times 100$$

(3) $$\mathrm{Sorption}\;\left(\mu g/mm^{3}\right)=\frac{m_{2}-m_{3}}{V}$$

(4) $$\mathrm{Solubility}\;\left(\%\right)=\frac{m_{1}-m_{3}}{m_{1}}\;\times 100$$

(5) $$\mathrm{Solubility}\;\left(\mu g/mm^{3}\right)=\frac{m_{1}-m_{3}}{V}$$

*m*_1_ is the baseline mass (before immersion in water), *m*(t) is the mass during the immersion and at different periods, *m*_2_ is the final saturated mass, and *m*_3_ is the final desiccated mass. *V* is the volume of the specimen (cylinder shape) and is calculated by the following formula: πr^2^h, where h is the height or thickness.

### Hygroscopic expansion

**FIGURE 2:**
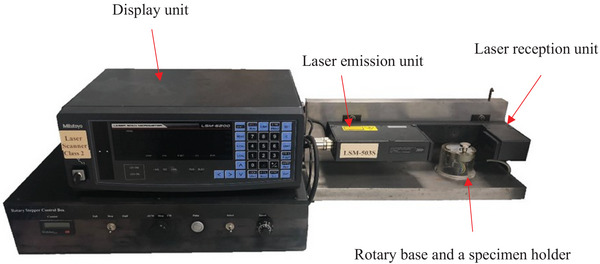
Laser scan micrometer (LSM) setup.

The same disk‐shaped specimens used for the water sorption and solubility measurement were concurrently used to measure their hygroscopic expansion. A custom‐built noncontact laser micrometer (Mitutoyo) was used to measure the dimensional changes of the specimens, described previously [2, 5, 11, 23, 24, 25, 26, 27, 28]. This method overcomes the limitations of nonuniform expansion, allowing a more accurate 3‐D assessment of hygroscopic expansion. The setup included an LSM‐503S measuring unit, an LSM‐6200 display unit, and a rotary stepper control box, which enabled the rotation of the specimen holder to allow for comprehensive diametral scanning (Figure 2). The resolution was 0.05 µm and the repeatability ± 0.2 µm [28]. The average diametral value was calculated from 360 individual readings. The initial mean diameter, d_1_, was taken before immersing the specimens in water. Subsequently, after each time interval, the diameter, d_2(t),_ was recorded.

To calculate diametral expansion (%), the following formula was used:

(6) $$\Delta d\;\;\left(\%\right)=\left(\frac{d_{2\left(t\right)}-d_{1}}{d_{1}}\right)\;\times 100$$

Volumetric expansion (%) was calculated, assuming isotropic expansion behavior, from the following formula:

(7) $$\Delta v\;\;\left(\%\right)=\left({\left(1+\frac{\Delta d}{100}\right)}^{3}-1\right)\;\times 100$$

### Zinc ion‐release analysis

Eighteen specimens (*n* = 3/group) were prepared in a similar way to the water sorption and solubility test. Each specimen was immersed in 10 mL of deionized water and incubated at 37°C for 1, 7, and 28 days. At each time interval, 9.7 mL of water was collected from each specimen and replaced with fresh deionized water. The collected water of each specimen was acidified with 0.3 mL of nitric acid (HNO₃) and filtered through a polyether sulfone (PES) with a pore size of 0.2 µm. The concentrations of Zn^2^⁺ ions were then determined using Inductively Coupled Plasma—Mass Spectrometry (ICP‐MS, Agilent 7700, Agilent Technologies). The method's limit of detection (LOD) and limit of quantification (LOQ) were 0.2 ppb and 5 ppb, respectively.

### Statistical analysis

The Shapiro–Wilk test was conducted to check if the data were normally distributed using SPSS statistical software (SPSS Statistics). Levene's test was used to evaluate the homogeneity of variances. One‐way ANOVA, followed by Tukey's post hoc test (*α* = 0.05), was conducted to determine any differences in hygroscopic characteristics between control and different n‐ZnO concentrations.

## RESULTS

### Water sorption and solubility

Table 3 shows the sorption and solubility for groups with increasing concentrations of n‐ZnO. The control group (ZnO at 0%) exhibited the highest water sorption and solubility values, which were 28.7 µg/mm^3^ and 2.1 µg/mm^3^, respectively. On the other hand, adding n‐ZnO led to significantly lower sorption and solubility when compared with the control group. Notably, groups with 2 wt.% of n‐ZnO showed a low sorption (27.0 µg/mm^3^) and negative solubility (−0.4 µg/mm^3^). However, no statistically significant differences were observed among the groups containing n‐ZnO except for the group with ZnO at 1 wt.%.

**TABLE 3:** Mean values (SD) of water sorption and solubility after final desiccation at 168 days.

Groups (wt.%)	Sorption (µg/mm^3^)	Solubility (µg/mm^3^)	Sorption (%)	Solubility (%)
Control (ZnO at 0 wt.%)	28.7 (0.6) ^A^	2.1 (0.3) ^A^	1.66 (0.03)	0.12 (0.02)
ZnO at 1 wt.%	28.3 (0.4) ^A,B^	1.4 (0.3) ^A,B^	1.61 (0.03)	0.08 (0.02)
ZnO at 2 wt.%	27.0 (0.2) ^C^	−0.4 (0.6) ^C^	1.52 (0.01)	−0.02 (0.03)
ZnO at 3 wt.%	27.9 (0.7) ^A,B,C^	0.4 (0.3) ^C,D^	1.55 (0.05)	0.02 (0.01)
ZnO at 4 wt.%	26.9 (0.6) ^C^	0.5 (0.4) ^D^	1.48 (0.03)	0.03 (0.02)
ZnO at 5 wt.%	27.5 (0.3) ^B,C^	0.8 (0.5) ^B,D^	1.50 (0.01)	0.04 (0.02)

*Note*: Similar superscript UPPERCASE letters indicate homogenous subsets within each column (*p* > 0.05). Specimens were immersed in deionized water at 37°C for 168 days and then desiccated for 168 days in an incubator.

**FIGURE 3:**
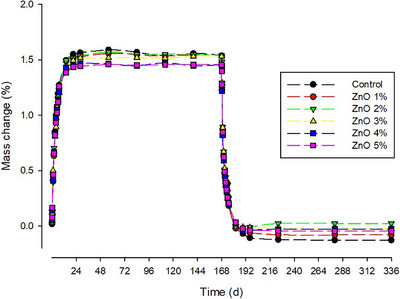
Mass change (%) over a period of 336 days for specimens with different n‐ZnO concentrations.

Figure 3 shows the percentage mass change over a 1‐year period. All groups exhibited a sharp increase within the first 3 weeks, followed by a plateau for the rest of the immersion period. As for the sorption, a marked decrease was observed during the first month; after that, the values stabilized.

### Hygroscopic expansion

Table 4 presents the dimensional changes, diametral and volumetric, for groups with increasing concentrations of n‐ZnO. The control group had the lowest expansion, with diametral expansion of 0.54% and volumetric expansion of 1.63%. In contrast, the addition of n‐ZnO led to a progressive increase in expansion as the concentration increased, peaking at 3 wt.% of n‐ZnO with a volumetric expansion of 1.87%. However, this trend did not persist for groups with 4 and 5 wt.% n‐ZnO. Statistical analysis shows that only the groups with 3 and 5 wt.% n‐ZnO had significant differences compared with the control group, while no significant differences were observed among the n‐ZnO‐containing groups.

**TABLE 4:** Mean values (SD) of diametral and volumetric expansion (%) after immersion in deionized water at 37°C for a period of 6 months.

Groups (wt.%)	Diametral expansion (%)	Volumetric expansion (%)
Control (ZnO at 0 wt.%)	0.54 (0.03) ^A^	1.63 (0.08) ^A^
ZnO at 1 wt.%	0.55 (0.05) ^A,B^	1.65 (0.14) ^A,B^
ZnO at 2 wt.%	0.59 (0.02) ^A,B^	1.77 (0.06) ^A,B^
ZnO at 3 wt.%	0.62 (0.05) ^B^	1.87 (0.15) ^B^
ZnO at 4 wt.%	0.59 (0.05) ^A,B^	1.78 (0.14) ^A,B^
ZnO at 5 wt.%	0.61 (0.01) ^B^	1.83 (0.04) ^B^

*Note*: Similar superscript UPPERCASE letters indicate homogenous subsets within each column (*p* > 0.05).

**FIGURE 4:**
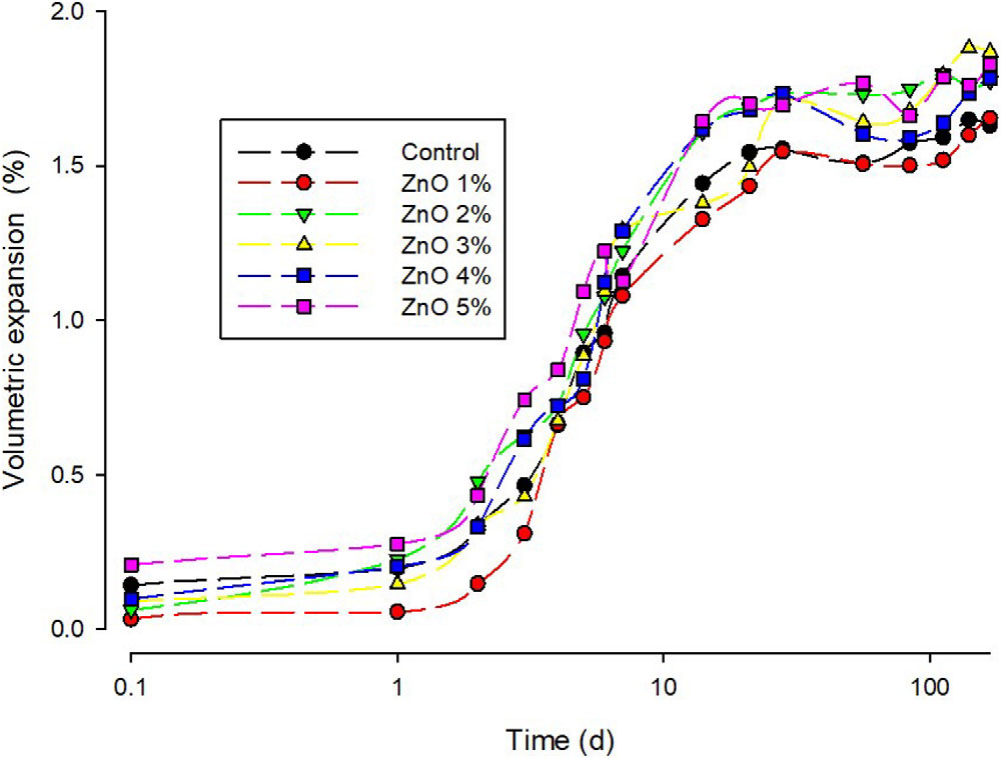
Volumetric expansion (%) over a period of 168 days for specimens with different n‐ZnO concentrations (logarithmic scale).

Figure 4 illustrates the cumulative volumetric expansion throughout the study period, showing a rapid increase in expansion within the first 10 days, followed by a gradual increase until reaching a maximum after 168 days.

### Zinc ion‐release analysis

**FIGURE 5:**
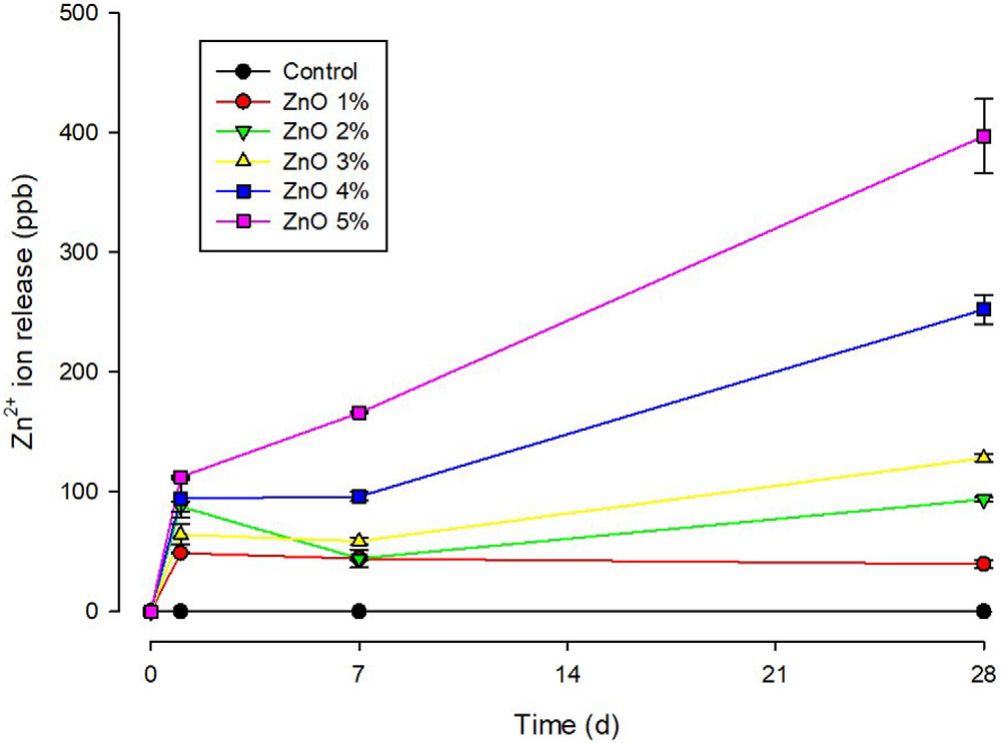
Zn^2^⁺ ion release analysis over a period of 28 days for specimens with different concentrations of n‐ZnO. The error bars represent the standard error of the mean.

Table 5, and illustrated in Figure 5, shows the results of Zn^2^⁺ ion release for groups with increasing concentrations of n‐ZnO. The cumulative release of Zn^2^⁺ increased progressively with higher concentrations of n‐ZnO. The highest Zn^2^⁺ ion release was observed in the ZnO at 5 wt.% group (675.1 ppb), followed by the 4 wt.% (442.7 ppb) and 3 wt.% (251.2 ppb) groups, with statistically significant differences between them.

**TABLE 5:** Mean values (SD) of Zn^2+^ concentrations after immersion in deionized water at 37°C for a period of 1, 7, and 28 days.

Groups (wt.%)	1 day	7 days	28 days	Cumulative ion release
Control (ZnO at 0 wt.%)	0.0	0.0	0.0	0.0 ^A^
ZnO at 1 wt.%	48.8 (2.2)	44.3 (22.2)	39.6 (10.3)	132.7 (14.7) ^B^
ZnO at 2 wt.%	87.7 (12.9)	44.4 (5.1)	93.7 (5.7)	225.8 (20.9) ^B^
ZnO at 3 wt.%	64.3 (24.9)	58.8 (9.5)	128.1 (8.7)	251.2 (43.1) ^B^
ZnO at 4 wt.%	94.3 (47.7)	96.1 (11.4)	252.3 (36.7)	442.7 (90) ^C^
ZnO at 5 wt.%	112.4 (3.4)	165.8 (2.6)	396.9 (93.2)	675.1 (95) ^D^

*Note*: Similar superscript UPPERCASE letters indicate homogenous subsets within the column (*p* > 0.05). Values are in (ppb).

The release of Zn^2^⁺ was initially rapid, with the amount released on the first day being nearly equivalent to that measured after 1 week, which shows a notable decrease in the release rate over the subsequent period.

## DISCUSSION

This study investigated the effect of adding n‐ZnO into a formulated RBC on the hygroscopic characteristics of specimens stored in deionized water at 37°C. A statistically significant difference was found when comparing the control group (without n‐ZnO) with groups that contained higher concentrations of n‐ZnO, leading to the rejection of the first and third null hypotheses. However, the second null hypothesis was only partially rejected, as some of the groups containing n‐ZnO did not exhibit a significant difference in hygroscopic expansion compared with the control.

In our study, all groups met the property requirement suggested by ISO 4049, even after prolonged storage, with water sorption and solubility values remaining below 40 and 7.5 µg/mm^3^, respectively [22]. This indicates that the material has acceptable stability and is capable of handling a moist environment [20]. The stability of our specimen could be related to the lower concentration of TEGDMA and the higher concentrations of Bis‐GMA and UDMA used, as TEGDMA exhibits greater sorption. A study conducted by Sideridou et al. [29] shows that TEGDMA absorbed more water (69.51 µg/mm^3^) when compared with Bis‐GMA (33.49 µg/mm^3^) and UDMA (29.46 µg/mm^3^). This is due to the presence of additional hydrophilic groups in TEGDMA that readily form hydrogen bonds with water, as well as the increased heterogeneity of its network resulting from primary cyclization [30].

Adding n‐ZnO led to a significant reduction in sorption and solubility values. However, our findings apparently contradict those of other studies, which reported an increase in their values following the addition of n‐ZnO [20, 21]. Brandão et al. [20] explained that the observed increases in their values after the addition of n‐ZnO are unexpected, as ZnO is insoluble in water. They suggested that this could be related to the degree of conversion of their specimen, as n‐ZnO addition reduced it [20]. This explanation may not apply to our findings. In our current study, we did not measure DC; however, our previous investigation, which evaluated up to 2 wt.% of n‐ZnO, found no decrease in DC from adding n‐ZnO, and DC remained between 77% and 79%, as shown in Table 2 [16]. Another possible reason for our result could be related to the filler/resin ratio [7, 31]. In our mixture, the polymer matrix accounted for approximately 40 wt.%. However, after the addition of n‐ZnO, the polymer loading decreased, resulting in lower sorption and solubility values. This is because the addition of fillers (n‐ZnO) results in a lower volume of the absorbing polymer since water sorption is a phenomenon primarily linked to the polymeric phase [31].

Various methods have been used to measure hygroscopic expansion, including Archimedes’ principle, optical scanning, contact profilometry, electronic and precision laser micrometers, and microscopes [2, 32]. This study assessed volumetric expansion using a laser scan micrometer. This offers a more reliable and reproducible measurement of small dimensional changes than the Archimedes method, which can be affected by the solubility of RBC [5, 28].

Groups containing higher concentrations of n‐ZnO exhibited higher expansion than the control group, despite having lower sorption and solubility. This outcome is unexpected, as the addition of n‐ZnO reduced the concentration of the monomer mixture in our study, and the primary factor influencing hygroscopic expansion is the polymer network [11, 27]. One possible explanation for this phenomenon is related to the diffusion of water into the resin system. Typically, when water diffuses into the RBC, it may either separate polymer chains by binding to hydrophilic groups or occupy the micro‐voids and the free volume between polymer chains. The latter phenomenon can occur without necessarily causing expansion [2, 11]. In our study, the addition of n‐ZnO particles to RBC may result in these particles occupying the micro‐voids in resin, leading to higher expansion values.

In our study, zinc ion release from the formulated RBC was increased as the n‐ZnO concentration increased, which is in agreement with several studies [21, 33]. The release was rapid after 1 day of immersion in water, but gradually slowed in the subsequent period. For instance, after the immersion of ZnO at 3 wt% group in water for 1 day, the zinc ion release was 64.3 ppb, whereas, after 1 week of immersion, the release was 58.8 ppb. A similar trend was observed in the Choi et al. study [21]; the release of zinc ions was high after 1 day of immersion, which was ∼3 ppm for RBCs with 4 wt% of ZnO. After the second day of immersion of similar specimens in water, the zinc ion release was ∼0.5 ppm [21]. The sustainable release of zinc ions is vital for maintaining efficient antibacterial activity. Several studies show that RBCs with ZnO can prevent bacterial growth by adding 1 wt.% or more, but when the material is aged for more than 1 week, the antibacterial effect diminishes [14, 18].

One limitation of this study is the use of a relatively low total filler content (60–65 wt.%), which is more characteristic of flowable RBC rather than conventional restorative materials. Although previous studies have reported increased water sorption at both high and low filler loadings [20, 21], the interaction between n‐ZnO and the resin matrix may differ under higher volumetric filler concentrations. Furthermore, the degree of conversion for higher n‐ZnO concentration was not investigated in this study or a previous study [16]. As such, the potential effects of increased filler loading or n‐ZnO on the hygroscopic characteristics and DC of n‐ZnO‐containing composites require further investigation in future studies.

This study demonstrated that the addition of n‐ZnO into RBC significantly influenced its hygroscopic characteristics. All groups met the ISO 4049 standards for sorption and solubility, with n‐ZnO addition leading to reduced values. Despite this, higher n‐ZnO concentrations unexpectedly resulted in increased hygroscopic expansion. Additionally, zinc ion release increased with higher n‐ZnO content and was most pronounced after 1‐day immersion in water. These findings suggest that the material exhibits promising long‐term stability in the oral environment, particularly in regard to sorption and solubility. Also, the zinc ion release from these formulations may contribute to their antibacterial effects. Although the observed increase in hygroscopic expansion at higher n‐ZnO concentrations is a potential concern, it may also offer a partial compensatory effect against polymerization shrinkage, which may help in internal stress relaxation.

## AUTHOR CONTRIBUTIONS

**Conceptualization**: Abdulaziz Alayed, Nikolaos Silikas, and David C. Watts. **Investigation**: Abdulaziz Alayed. **Methodology**: Abdulaziz Alayed, Nikolaos Silikas, and David C. Watts. **Formal analysis**: Abdulaziz Alayed. **Writing—original draft**: Abdulaziz Alayed. **Writing—review & editing**: Nikolaos Silikas and David C. Watts.

## CONFLICT OF INTEREST STATEMENT

The authors declare no conflict of interest.
